# A six-gene expression signature in peripheral blood mononuclear cells effectively diagnoses osteoarthritis

**DOI:** 10.3389/fmed.2025.1632348

**Published:** 2025-10-15

**Authors:** Dong Yu, Wei Ding, Xiuru Xue, Zheng Zhang, Jinchang Meng, Bin Yang, Chunlin Liang, Guanghui Zhao, Xiangmao Bu, Wei Chen

**Affiliations:** ^1^Department of Library, Shandong Second Medical University, Weifang, China; ^2^Clinical Laboratory, Peking University People’s Hospital, Qingdao; Women and Children’s Hospital, Qingdao University, Qingdao, China; ^3^Joint Surgery, Peking University People’s Hospital, Qingdao; Women and Children’s Hospital, Qingdao University, Qingdao, China

**Keywords:** osteoarthritis, PBMC, RNA sequencing, diagnostic model, expression signature

## Abstract

**Introduction:**

Osteoarthritis (OA) is a heterogeneous whole-joint disease that inconveniences more than 500 million people worldwide. Early diagnostic methods for OA remain lacking. Peripheral blood mononuclear cells (PBMCs) are ideal sample sources for the early diagnosis of different diseases. However, only a few studies have reported on the role of PBMCs in the early diagnosis of OA.

**Methods:**

RNA sequencing was performed on PBMC samples from 27 patients with OA and 31 healthy controls. We integrated RNA sequencing data from our internal cohort and microarray data from external cohort to construct a diagnostic model of OA based on PBMC samples. The receiver operating characteristic (ROC) curve analysis was used to evaluate the diagnostic model in PBMC samples and synovial tissue.

**Results:**

In this study, we screened and constructed a six-gene diagnostic model consisted of the genes THBS1, USP36, GIMAP4, OSM, IL10, and HDC, which could effectively distinguish patients with OA from healthy controls. The ROC curve analysis showed that the area under curve (AUC) of this diagnostic model was 0.928 for our internal cohort and 0.915 for the external cohort, respectively. Interestingly, the gene expression model also had high accuracy (AUC = 0.910) for diagnosing patients with OA based on expression data from synovial tissue.

**Discussion:**

Given that related studies on several signature genes in our diagnostic model for OA are lacking, our study provides novel potential biomarkers for the early diagnosis of OA based on PBMC samples.

## Introduction

Osteoarthritis (OA) is a heterogeneous whole-joint disease that can cause pain and result in disability and premature work loss, affecting more than 500 million people worldwide (1, 2). Its prevalence is mainly attributed to aging, obesity, and joint injuries (1, 3). Although physical examination, radiographic indicators and biochemical markers have significantly advanced the diagnosis of OA, these conventional techniques might detect OA only at advanced stages, thereby limiting early detection (4, 5). Therefore, novel diagnostic methods are urgently needed for early detection of OA.

Peripheral blood mononuclear cells (PBMCs) are a large accessible sample source for developing early diagnostic methods for different diseases due to their non-invasiveness, ease of preparation, and rich content of molecular markers (DNA, RNA, and protein). PBMCs are also an important sample type investigated in OA. Studies involving PBMCs have been used in studies to characterize immune cell dysfunction (6, 7), molecular dysfunction (8, 9), and OA treatment (10, 11). However, few studies have comprehensively reported the role of PBMCs in the early diagnosis of OA.

In this study, we performed RNA sequencing on PBMC samples from 27 OA patients and 31 healthy controls. By integrating our sequencing data and a public external dataset, we further identified a six-gene expression signature that could effectively distinguish OA patients from healthy controls. Our six-gene diagnostic model consisted of THBS1, USP36, GIMAP4, OSM, IL10, and HDC, several of which have not previously been studied in OA. Moreover, the diagnostic model demonstrated high accuracy for distinguishing OA patients based on expression data of synovial tissue.

## Materials and methods

### Sample collection and RNA extraction

The blood samples were collected from 27 OA patients and 31 healthy controls at Peking University People’s Hospital, Qingdao, between December 2024 and January 2025 (Supplementary Table S1). Sample collection was approved by the Ethical Committees of Peking University People’s Hospital, and all participants signed the written informed consent.

PBMCs were isolated from blood within 2 h by density gradient centrifugation using Ficoll solution (Sigma-Aldrich, United States) and subsequently stored at −80 °C until RNA extraction. Total RNAs were extracted from PBMC samples with TRIzol LS reagent (Thermo Fisher Scientific, United States), and quality check was measured by Qubit 3.0 (Thermo Fisher Scientific, United States) and Agilent 2100 Bioanalyzer (Agilent Technologies, United States).

### Library construction, mRNA sequencing, and analysis

mRNAs were isolated using oligo-dT method with VAHTS mRNA Capture Beads (Vazyme, China), and mRNA sequencing library was prepared according to the protocol of VAHTS Universal V6 RNA-seq Library Prep Kit for Illumina (Vazyme, China) at Genesky Biotechnologies Inc., Shanghai, China. The library was evaluated with Qubit 3.0 (Thermo Fisher Scientific, United States) and Agilent 2100 Bioanalyzer (Agilent Technologies, United States). Sequencing was performed with paired-end 150 bp on the Illumina NovaSeq 6000 platform (Illumina, United States).

Raw sequencing reads (6 G data per sample, approximately 2×) were evaluated for quality using FastQC (version 0.11.8). Sequencing primers, and low-quality reads, and remaining reads shorter than 40 bp were removed. Clean reads (Q30 >94.22%, approximately 25.86 million reads per sample) were mapped to the human reference genome, hg38, achieving an average of 97.72% aligned bases per sample, using STAR (version 2.7.10b). StringTie (version 1.3.5) was used for transcript assembly and quantification. The differentially expressed genes (DEGs) between patients with OA and healthy controls were identified by DESeq2 package (version 1.10.1) with the absolute value of log2 (fold change) > 1 and *p*-value < 0.05.

### Gene expression dataset and analysis

The gene expression data of 139 PBMC samples from 106 OA patients and 33 healthy controls were obtained from the GSE48556 dataset of the Gene Expression Omnibus (GEO) database.^1^ The healthy controls were sex- and age-matched with the patients. Expression profiles were analyzed by GPL6947 platform (Illumina HumanHT-12 V3.0 expression beadchip). The DEGs with 1.2-fold change and *p*-value <0.05 were identified between OA patients and healthy controls using Limma (linear models for microarray data, version 3.40.6) in R programming language. The gene expression data of 20 synovial tissue samples from 10 OA patients and 10 healthy controls were also obtained from the GSE55235 dataset of GEO for the validation of the diagnostic model.

### Random forest analysis

The internal RNA-seq data were used to screen candidate genes by the random forest analysis using randomForest (version 4.7.1.1), which calculates an importance score of each gene. Genes with mean decrease accuracy >2.0 were selected as candidate genes.

### Reverse transcription-quantitative polymerase chain reaction

RNA extraction was conducted as described above, and cDNA was synthesized using 40 randomly selected OA and healthy control samples by Reverse Transcriptase M-MLV (Takara, Japan) in accordance with instructions. In addition, reverse transcription-quantitative polymerase chain reaction (RT-qPCR) was performed with SYBR^®^ Premix Ex Taq^™^ II (Takara, Japan). Glyceraldehyde-3-phosphate dehydrogenase (GAPDH) was used as an endogenous control for the candidate genes. All primers of these genes were listed in Supplementary Table S2. The relative expression of each gene between OA and healthy control samples was compared using 2^−ΔΔCt^ method, with ∆Ct = Ct_gene_ − Ct_GAPDH_.

### Gene pathway analysis

Kyoto Encyclopedia of Genes and Genomes (KEGG) analysis was conducted to identify significant pathways enriched in DEGs between OA patients and healthy controls using clusterProfiler package (version 4.4.4) in R (12). Pathways with adjusted *p*-value <0.05 (Bnejamini & Hochberg) were considered as significantly enriched.

### Statistical analysis

All data processing was performed using R 4.2.1 software. The Wald test was used for the differential expression analysis of mRNA sequencing between OA and control samples. The Wilcoxon rank–sum test was adopted for the differential analysis of the six genes between two groups from different datasets, as well as for the RT-qPCR analysis of these genes. Multifactor logistic regression analysis was implemented to determine independent predictive models, and receiver operating characteristic (ROC) curve was used to evaluate the distinguishing effect of the model by employing package “pROC” (version 1.18.0) in R. Uniform Manifold Approximation and Projection (UMAP) was performed to analyze the mRNA sequencing data with package “UMAP” (version 0.2.10.0). The results were visualized by ggplot2 (version 3.4.4). All statistical results with a *p*-value <0.05 were considered to be significant.

## Results

### Differential expression analysis of PBMC samples from OA patients and controls in internal cohort

**Figure 1:**
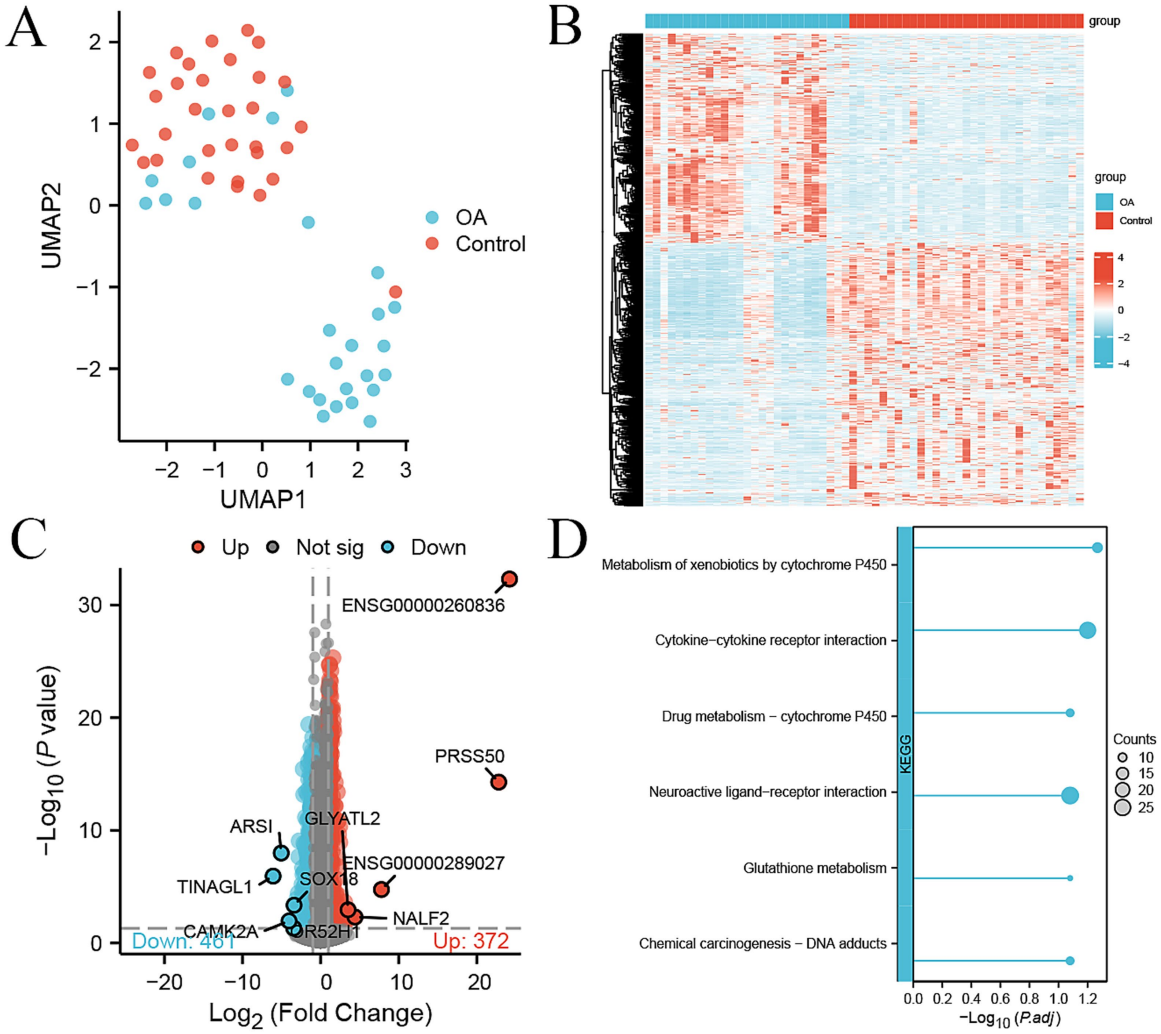
DEGs between 27 OA and 31 control samples in the internal cohort. **(A)** UMAP plot showing the dimension reduction of all genes in 27 OA and 31 control samples. **(B)** Heatmap of 833 DEGs between OA and control samples, with expression levels normalized by *z*-score. **(C)** Volcano plot of 372 up-regulated (red) and 461 down-regulated genes (green) between OA and control, with the top five genes labeled. **(D)** KEGG pathway enrichment analysis of DEGs; the size of the dot represents the count of DEGs.

The UMAP showed that most of 27 OA and 31 control samples were clearly separated, indicating that the expression profiles in the PBMCs of OA patients had changed compared to those of the control (Figure 1A). A total of 833 DEGs were identified in PBMCs between OA patients and healthy controls, with 372 up-regulated and 461 down-regulated genes [|log_2_ (fold change)| >1, *p*-value <0.05] in the OA group (Figure 1B). The top up-regulated genes were ENSG00000260836, PRSS50, ENSG00000289027, NALF2, and GLYATL2, while the top down-regulated genes were TINAGL1, ARSI, CAMK2A, OR52H1, and SOX18 (Figure 1C). The pathway analysis of these DEGs showed that cytochrome P450-related pathways, cytokine–cytokine receptor interaction, and glutathione metabolism were significantly enriched (Figure 1D).

### Differential expression analysis of PBMC samples from OA patients and controls in external cohort

**Figure 2:**
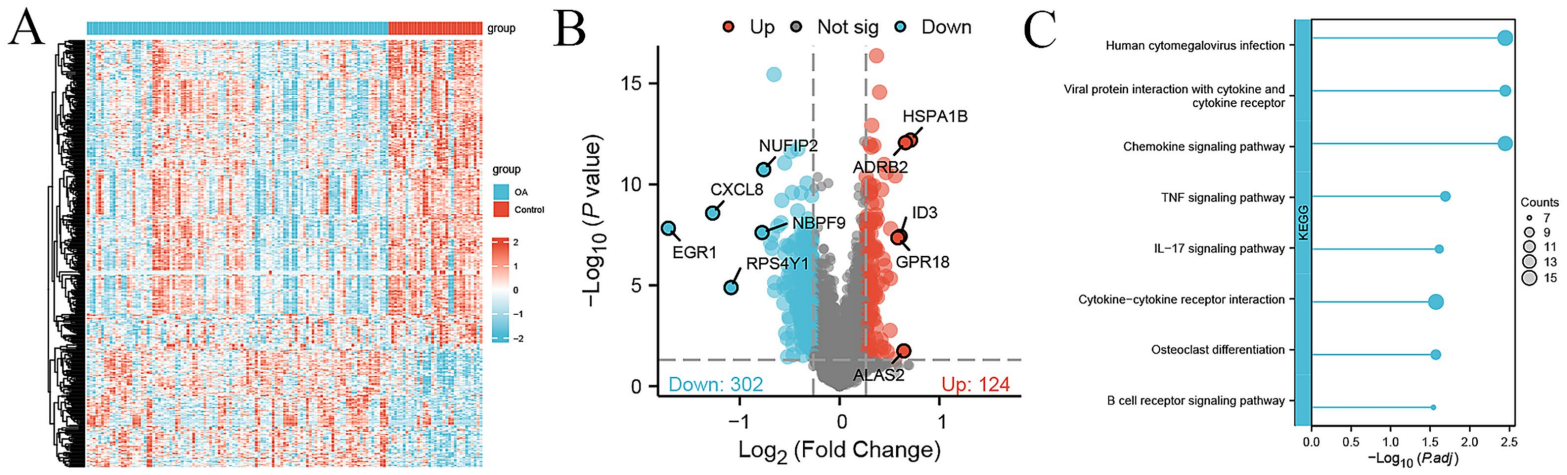
DEGs between 106 OA and 33 control samples in the external cohort. **(A)** Heatmap of 426 DEGs between 106 OA and 33 control samples, with expression levels normalized by *z*-score. **(B)** Volcano plot showing 124 up-regulated (red) and 302 down-regulated genes (green) between OA and control, with top the top genes labeled. **(C)** KEGG pathway enrichment analysis of DEGs; the size of the dot represents the count of DEGs.

We used the GSE48556 dataset from the GEO database to compare differences in gene expression in PBMC samples from 106 OA patients and 33 healthy controls. A total of 426 genes were differentially expressed (124 up-regulated and 302 down-regulated) with fold change >1.2 and *p*-value <0.05 (Figure 2A). The top up-regulated genes included HSPA1B, ADRB2, ALAS2, ID3, and GPR18, while the top down-regulated genes included EGR1, CXCL8, RPS4Y1, NBPF9, and NUFIP2 (Figure 2B). The KEGG pathway enrichment analysis of the above DEGs showed that a series of immunity-related pathways were significantly enriched, including chemokine signaling pathway, IL-17 signaling pathway, and cytokine−cytokine receptor interaction. In addition, osteoclast differentiation was significantly enriched (Figure 2C).

### Identification of signature genes for predicting OA patients based on expression data of PBMC samples

**Figure 3:**
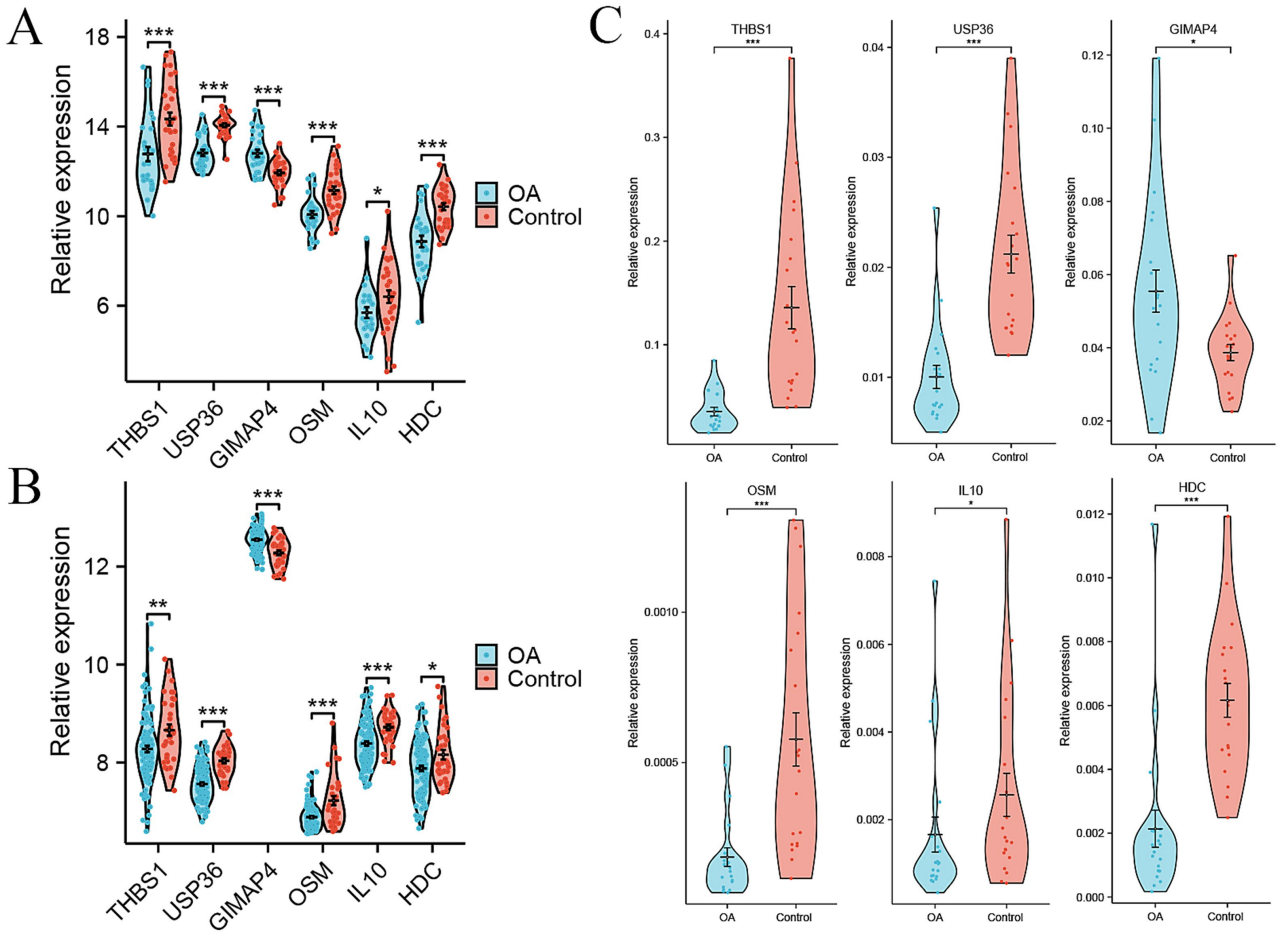
Signature genes for distinguishing OA patients from healthy controls based on expression data of PBMC samples. **(A)** Violin plots showing the relative expression levels of six signature genes between OA and control in internal cohort. **(B)** Violin plots showing the relative expression levels of six signature genes between OA and control in external GSE48556 cohort. **(C)** Relative expression levels of six signature genes between OA and control by RT-qPCR. ^*^p < 0.05, ^**^p < 0.01, and ^***^p < 0.001.

**Figure 4:**
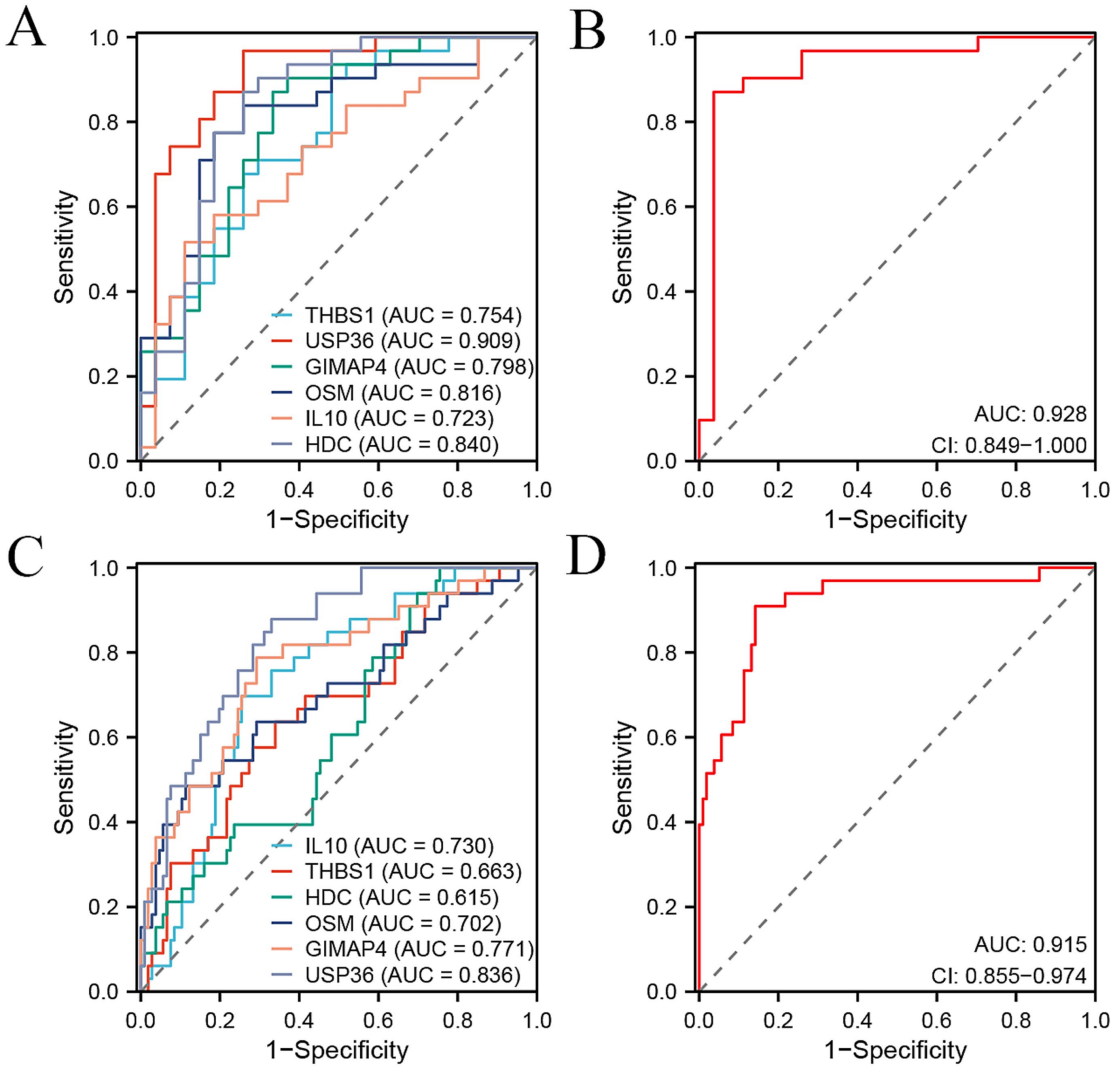
ROC curve analysis of six signature genes and their combination signature for distinguishing OA patients from the controls in two cohorts. **(A,B)** ROC curve showing the predictive efficiency of six signature genes **(A)** and their combination **(B)** for distinguishing OA patients from healthy controls in our internal cohort. **(C,D)** ROC curve showing the predictive efficiency of six signature genes **(C)** and their combination **(D)** for distinguishing OA patients from healthy controls in external GSE48556 cohort.

To identify the candidate signature genes for distinguishing OA patients from healthy controls based on gene expression data of PBMC samples, we firstly performed random forest analysis on our internal PBMC data and identified several candidate genes for further evaluation. We selected the top nine genes (PPP1R16B, MRPS31, DDIT4L, GRIN2C, PHKG1, UGDH, GTPBP1, LRRC4B, and SMIM8, with mean decrease accuracy >2.0) for ROC analysis (Supplementary Figure S1A). In our internal cohort, these genes showed significant differences between OA and control samples (*p* < 0.05, Supplementary Figure S1B), and each gene could accurately distinguish OA samples from control samples with a high area under curve (AUC) value (Supplementary Figure S1C). However, in the external PBMC cohort, some genes (GRIN2C, LRRC4B, GTPBP1, PHKG1, and UGDH) did not show significant differences between OA and control samples (Supplementary Figure S1D), and the AUC values of most genes, except for MRPS31, were less than 0.7 (Supplementary Figure S1E). Therefore, we did not select these genes identified by random forest analysis for model construction. As an alternative, we obtained intersecting DEGs in the internal cohort and external cohort, including 15 genes, namely CXCR5, THBS1, CXCR3, CEMIP2, USP36, GIMAP4, EAF2, GNG11, OSM, TFPI, IL10, CLEC1B, SH3BGRL2, PVALB, and HDC (Supplementary Table S3). However, only six genes THBS1, USP36, GIMAP4, OSM, IL10, and HDC demonstrated consistent changes in the internal and external cohort. GIMAP4 was up-regulated, while the other five genes were down-regulated in OA patients compared to healthy controls (Figures 3A,B). Therefore, we screened these six genes as candidate signature genes for distinguishing OA patients and healthy controls. The RT-qPCR results validated that the expression of GIMAP4 was significantly up-regulated, and those of THBS1, USP36, OSM, IL10, and HDC were consistently down-regulated in OA patients compared to healthy controls (Supplementary Table S4 and Figure 3C). The AUC of ROC curve was used to evaluate the diagnostic efficacy of six genes for predicting OA on the basis of PBMC samples. We obtained AUC values of 0.754 for THBS1, 0.909 for USP36, 0.798 for GIMAP4, 0.816 for OSM, 0.723 for IL10, and 0.840 for HDC in the internal cohort (Figure 4A). Our combined six-gene model had an AUC of 0.928, with a sensitivity of 0.871 and specificity of 0.963 for the internal cohort (Figure 4B). We obtained AUC values of 0.663 for THBS1, 0.836 for USP36, 0.771 for GIMAP4, 0.702 for OSM, 0.730 for IL10, and 0.615 for HDC in the external cohort (Figure 4C). Our combined six-gene model had an AUC of 0.915, with a sensitivity of 0.909 and specificity of 0.858 for the internal cohort (Figure 4D). These results suggested that our six-gene diagnostic model had high accuracy for predicting patients with OA based on the expression data of PBMC samples. The screening strategy employing intersecting DEGs from different detection platform might yield robust candidate results, as it ensures the consistency of genes across different platforms or cohorts and reduces biases arising from platform differences. In addition, considering that USP36 and GIMAP4 have not been reported in OA-related studies, our current analysis could identify novel signature genes for diagnosing patients with OA.

### Validation of the diagnostic model for predicting OA based on synovial tissue samples

**Figure 5:**
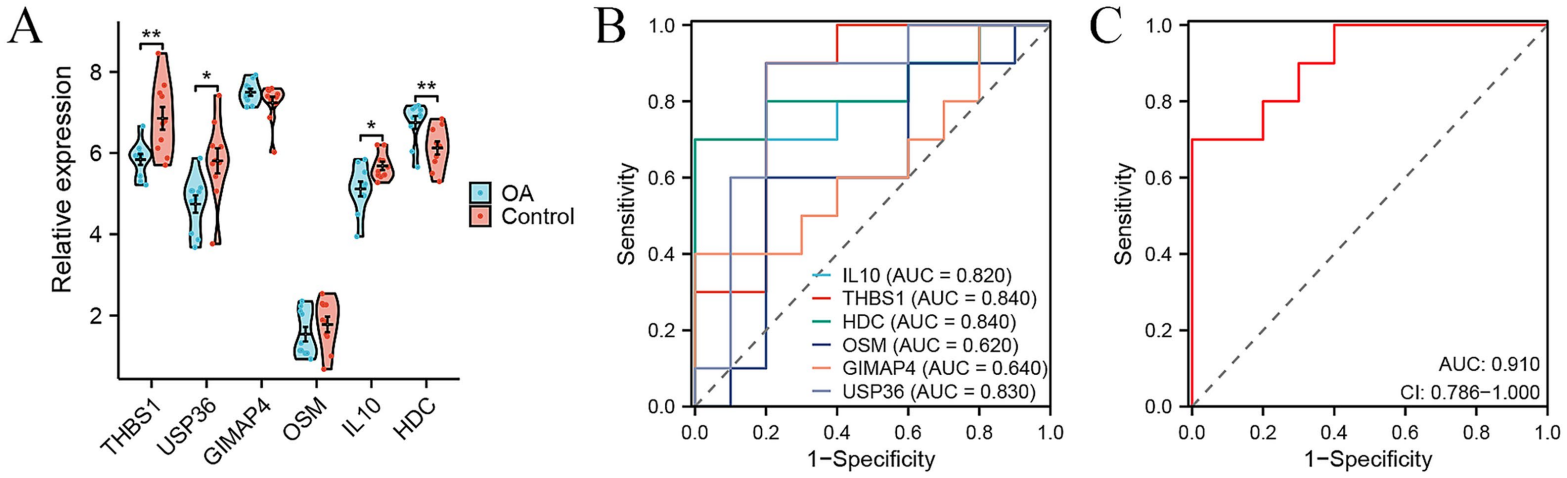
Signature genes for distinguishing OA patients from healthy controls based on expression data of synovial tissue samples. **(A)** Violin plots showing the relative expression levels of six signature genes between OA and control in external GSE55235 cohort. **(B,C)** ROC curve showing the predictive efficiency of six signature genes **(B)** and their combination **(C)** for distinguishing OA patients from healthy controls in external GSE55235 cohort. ^*^p < 0.05, ^**^p < 0.01, and ^***^p < 0.001.

To further validate the diagnostic efficacy of the six genes for distinguishing patients with OA from healthy controls in tissue samples, we analyzed the gene expression data of synovial tissue from 10 OA patients and 10 healthy controls in the GSE55235 dataset from GEO database. Most of these six genes, except for HDC, showed consistent changes or significances in PBMC samples and synovial samples (Figure 5A). We obtained AUC values of 0.840 for THBS1, 0.830 for USP36, 0.640 for GIMAP4, 0.620 for OSM, 0.820 for IL10, and 0.840 for HDC in GSE55235 (Figure 5B). Our combined six-gene model had an AUC of 0.910, with a sensitivity of 0.7 and specificity of 1 (Figure 5C). These results validated that our six-gene diagnostic model also had high accuracy for predicting patients with OA based on the gene expression data of synovial tissue. Therefore, these genes serve as ideal biomarkers for the early diagnosis of OA.

## Discussion

Although joint imaging is still the primary method for diagnosing and monitoring of OA in clinical practice (13), early detection of the disease remains limited. Previous studies reported several related diagnosis models for OA based on gene expression information. However, most of them had relatively small sample size, which affects the diagnostic efficacy. For instance, Zeng’s et al. (14) study only contained 24 patients with OA and 24 controls from public data, Chen’s study included 28 patients with OA and 26 controls (15). Liang et al. (16) found APOLD1 and EPYC as diagnostic genes from 20 patients with OA and 20 controls.

In addition, other studies constructed the diagnosis model of OA that relied on the external public data entirely, including the above studies with small sample size. For instance, Tu et al. (17) reported the role of arachidonic acid metabolism-related genes for OA diagnosis based on public datasets. Similar studies included the diagnostic model of anoikis-related genes (18), macrophage-associated genes (19), and lactate metabolism-related gene signature (20). Unlike these, our study combined our internal cohort (27 OA patients and 31 controls) and public cohort (106 OA patients and 33 controls) with more samples to develop a six-gene model for OA diagnosis, which had better predicting performance. Notably, we not only established a prediction model in PBMC samples but also conducted validation in synovial tissue. We considered that diverse types of immune cells in PBMC might migrate to synovial tissue, thereby participating in the onset and progression of OA. For example, CCR2^+^ cells were abundant in human synovium of OA and that blockade of CCL2/CCR2 signaling markedly attenuated macrophage accumulation, synovitis, and cartilage damage in mouse OA (21). Another study showed that end-stage OA knees (including synovial samples) were characterized with an increased CD4^+^ T cell polarization toward activated Th1 cells and cytokine secretion (including anti-inflammatory IL10, a component of our six-gene diagnostic model) compared to peripheral blood samples (22). These findings indicated that there were multiple relations between immune cells and synovial tissue, which had significant impact on OA. Therefore, we selected PBMC and synovial tissue samples to evaluate our six-gene model for OA diagnosis.

Another advantage of our study was using blood or PBMC samples, which could realize early and non-invasive diagnosis for OA based on the expression signatures of blood cells. However, most of previous related studies constructed diagnostic models using synovial or cartilage tissue samples (16–19, 23, 24), which were not conducive to early diagnosis and obtained by invasive methods.

In our study, a six-gene diagnostic model was identified in OA, including THBS1, USP36, GIMAP4, OSM, IL10, and HDC. Among them, USP36 or GIMAP4 have not been previously reported in OA, indicating their potential as novel targets and biomarkers for OA. Importantly, this study validated that THBS1 partly mediated the cartilage protective effect by reducing inflammation in OA (25). In addition, THBS1 was reported to be a shared biomarker between myocardial infarction and OA (26). The protein level of THBS1 was also significantly differential in synovial fluid between 24 patients with OA and 24 healthy persons (27). OSM and IL10 were widely studied in OA. Oncostatin M (OSM) was found overexpressed in knee OA, and Notch signaling inhibited OSM-induced cell proliferation and differentiation (28). As a member of IL6 family, OSM was demonstrated to drive an inflammation phenotype in knee OA (29). Anti-inflammatory cytokines, including IL10, have been widely discussed in the pathogenesis of OA (30). Importantly, targeting IL10 might be an effective therapy for OA, potentially reducing pain (31) and alleviating cartilage degeneration (32). Histidine decarboxylase (HDC) could stimulate the proliferation of human articular chondrocytes, and its expression by chondrocytes was demonstrated in OA cartilage (33, 34). In addition, HDC was found as a prototypical mast cell marker in OA synovial cells by single-cell RNA sequencing (35). Therefore, most genes played important roles in the pathogenesis or therapy of OA. The combined gene model outperformed individual genes in distinguishing OA patients from healthy controls in each cohort, as the combination of these genes could capture the molecular characteristics of OA from different dimensions. Therefore, it is necessary to develop the combined gene model as the predictive tool for OA.

However, the study had some limitations. Firstly, although we collected 58 PBMC samples from 27 patients with OA and 31 healthy controls, the sample count should be further amplified to validate the efficacy of our six-gene diagnostic model. Secondly, the topic of this study was constructing a diagnostic model based on gene expression signatures. The RNA or protein expression levels of these six genes should be further validated in PBMC and synovial or cartilage tissue samples. Thirdly, some signature genes, such as USP36 and GIMAP4, have not be previously studied in OA. Their molecular functions could be further explored in OA, for instance, overexpression or knockdown of USP36/GIMAP4 *in vitro* could be used to assess effects on cell proliferation, apoptosis, or the secretion of inflammatory factors in synovial cells or chondrocytes.

In conclusion, this study integrates the internal and external RNA data of PBMC samples to construct a diagnostic model for predicting OA, which could effectively distinguish patients with OA from healthy controls by six-gene expression signatures of PBMC or synovial tissue samples. It provides important value for the early diagnosis of OA based on blood-derived samples, and finds some potential biomarkers and targets for OA.

## Data Availability

The data that support the findings of this study are publicly accessible at https://bigd.big.ac.cn/gsa-human/browse/HRA011922.

## Footnotes

^1^ https://www.ncbi.nlm.nih.gov/geo/

## Supplementary material

SUPPLEMENTARY FIGURE S1 Signature genes screening and evaluation by random forest analysis in internal and external PBMC samples. **(A)** Random forest analysis showed the mean decrease accuracy of top genes. **(B)** Violin plots showed the relative expression levels of nine signature genes between OA and control in our internal cohort. **(C)** The ROC curve showed the predictive efficiency of nine genes for distinguishing OA patients from healthy controls in internal cohort. **(D)** Violin plots showed the relative expression levels of nine signature genes between OA and control in external GSE48556 cohort. **(E)** The ROC curve showed the predictive efficiency of nine genes for distinguishing OA patients from healthy controls in external GSE48556 cohort. ^*^*p* < 0.05, ^**^*p* < 0.01, and ^***^*p* < 0.001.
