# Supplementary figures and images for: A six-gene expression signature in peripheral blood mononuclear cells effectively diagnoses osteoarthritis
Source: Front Med (Lausanne). 2025 Oct 15;12:1632348. doi: 10.3389/fmed.2025.1632348 (PMC12568575; doi:10.3389/fmed.2025.1632348)

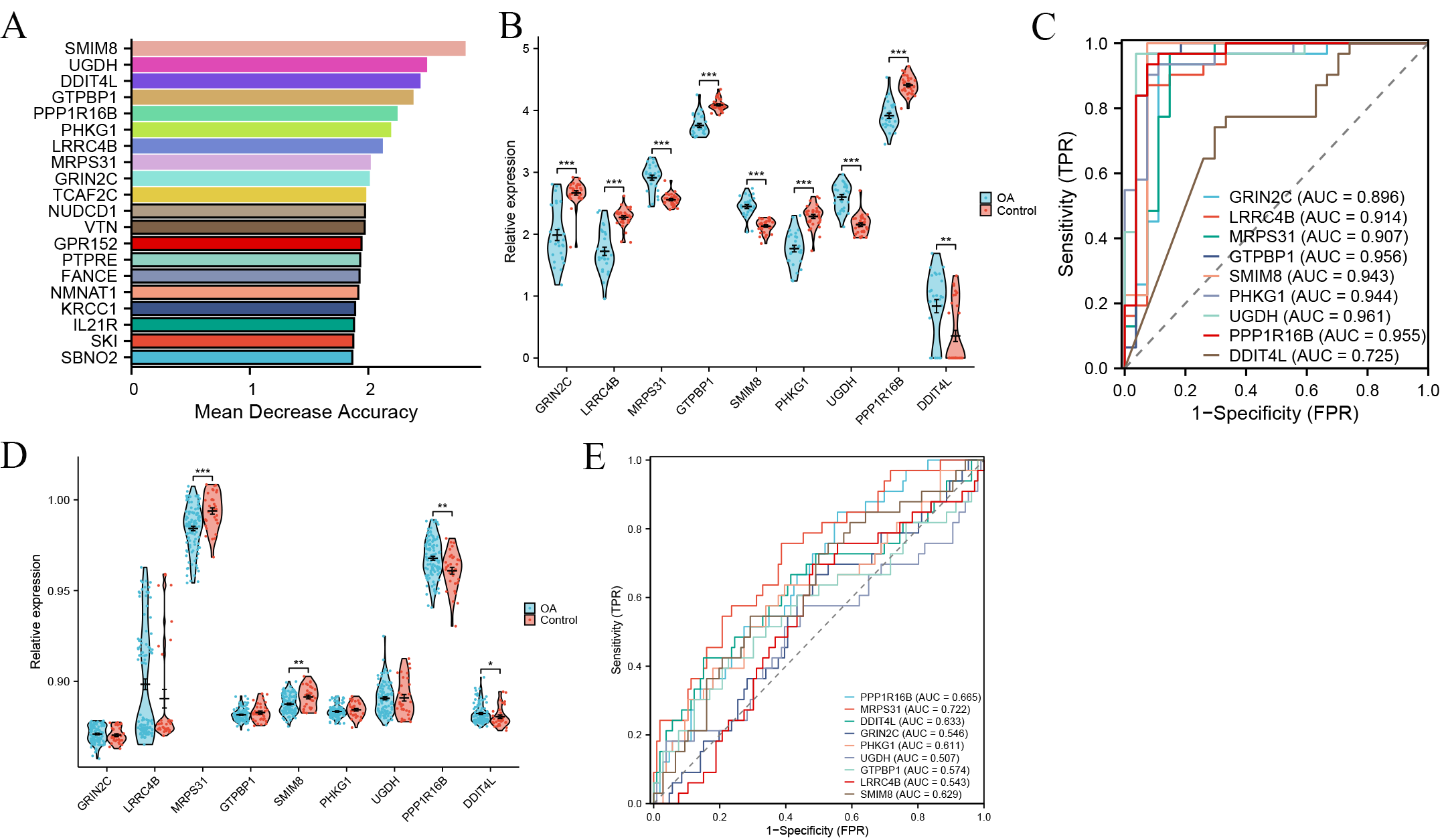

Supplement: SUPPLEMENTARY FIGURE S1 — Signature genes screening and evaluation by random forest analysis in internal and external PBMC samples. (A) Random forest analysis showed the mean decrease accuracy of top genes. (B) Violin plots showed the relative expression levels of nine signature genes between OA and control in our internal cohort. (C) The ROC curve showed the predictive efficiency of nine genes for distinguishing OA patients from healthy controls in internal cohort. (D) Violin plots showed the relative expression levels of nine signature genes between OA and control in external GSE48556 cohort. (E) The ROC curve showed the predictive efficiency of nine genes for distinguishing OA patients from healthy controls in external GSE48556 cohort. *p < 0.05, **p < 0.01, and ***p < 0.001. [file Image_1.TIF]
